# Pain threshold in adolescents with juvenile idiopathic arthritis and fibromyalgia

**DOI:** 10.1186/1546-0096-12-S1-P295

**Published:** 2014-09-17

**Authors:** Juliana Molina, Liana Sanches, Maria Teresa Terreri, Edson Amaro, Claudio A Len

**Affiliations:** 1 Pediatrics, UNIFESP, Brazil; 2 Brain Institute, Hospital Albert Einstein, São Paulo, Brazil

## Introduction

Pain is a frequent complaint in pediatric practice and is present in several chronic organic diseases, such as juvenile idiopathic arthritis (JIA). While JIA patients show symptoms such as inflammation of the joints and other structures, such as the heart and eyes, patients with idiopathic musculoskeletal pain (IMP) experience a painful condition that is not associated with presence of tissue injuries. Juvenile fibromyalgia (JFM), disorder characterized by recurrence of disabling pain, is a classic example IMP. This study shows preliminary data of a protocol for evaluation of brain activation using functional magnetic resonance imaging (fMRI) after a painful stimulus produced by pressure.

## Objectives

To evaluate and compare the pain threshold in adolescents with JIA and JFM that will be examined by fMRI scan.

## Methods

Twenty nine adolescents were divided into 3 groups: 10 adolescents with JFM, 9 adolescents with JIA and 10 healthy adolescents without complaints of pain.

Using a mechanical system, designed for experiments with fMRI, a series of discrete pressure stimulus were performed, with duration of 5 seconds, applied on the left thumb by a stiff rubber tube connected to a hydraulic piston, enabling a controlled and reproducible stimulation. Participants were asked to graduate the intensity of pain sensation evoked by an ascending series of pressure stimulus, until the subjective rating of pain reported was graded as 4 (four).

## Results

The amount of pressure used in the pressure stimulus was significantly different between groups (p = 0.0003). The pain threshold was lower in JFM group (mean pressure used = 3.70 kg/cm^2^), followed by the group of healthy adolescents (4.45 kg/cm^2^) and the JIA group (4.88 kg/cm^2^). All participants reported the same subjective pain rating 4 (four).

## Conclusion

Adolescents with JFM presentes a decrease in the threshold for pain, which was significantly lower when compared with adolescents with JIA with long history of organic pain.

## Disclosure of interest

None declared.

